# Acceleration of Ethanol Metabolism by a Patented *Bos taurus* Isolated Alcohol Degradation Protein (ADP) on Acute Alcohol Consumption

**DOI:** 10.3390/foods13193207

**Published:** 2024-10-09

**Authors:** Bun Tsoi, Huan Zhang, Chun-Pang So, Angel Ka-Kei Lam, Christina Chui-Wa Poon, Sek-Lun Law, Bing-Lou Wong, Sai-Wang Seto

**Affiliations:** 1 Department of Food Science and Nutrition, The Hong Kong Polytechnic University, Hong Kong, China; bun.tsoi@polyu.edu.hk (B.T.); zhanghuan.zhang@polyu.edu.hk (H.Z.);; 2 Research Centre for Chinese Medicine Innovation, The Hong Kong Polytechnic University, Hong Kong, China; 3 School of Professional and Continuing Education (HKU SPACE), The University of Hong Kong, Hong Kong, China; 4 Alcolear Limited, Fotan, New Territories, Hong Kong, China; 5 NICM Health Research Institute, Western Sydney University, Penrith, NSW 2751, Australia; 6 School of Biomedical Sciences, The University of Western Australia, Perth, WA 6009, Australia

**Keywords:** alcohol degradation, hangover, liver protection

## Abstract

Alcoholic beverages are among the most widely enjoyed leisure drinks around the world. However, irresponsible drinking habits can have detrimental effects on human health. Therefore, exploring strategies to alleviate discomfort following alcohol consumption would be beneficial for individuals who inevitably need to consume alcohol. In this study, three different models were used to determine the efficacy of a patented alcohol degradation protein (ADP) extracted from *Bos taurus* on ethanol metabolism. In an ethanol-challenged HepG2 cell model, ADP significantly protected the cell from ethanol-induced toxicity. Subsequently, results demonstrated that ADP significantly alleviated the effect of ethanol, as reflected by the increased distance and activity time of zebrafish during the testing period. Additionally, in a rat model, ADP promoted ethanol degradation at 1 and 2 h after ethanol consumption. Mechanistic studies found that ADP treatment increased ADH and ALDH activity in the gastrointestinal tract. ADP also exhibited potent antioxidation effects by lowering HO-1 expression in the liver. In conclusion, we believe that ADP is a promising product for relieving hangover symptoms after ethanol consumption, with demonstrated safety and effectiveness at the recommended dosage.

## 1. Introduction

Alcoholic beverages are widely consumed by people across diverse age groups and cultural backgrounds. Apart from leisure consumption, alcohol consumption is expected or considered obligatory in social or business settings. In addition to subjective drinking (voluntary consumption of alcoholic beverages by one’s own will), ethanol can also be produced in all organisms by several physiological pathways, such as fatty acid synthesis, glycerolipid metabolism and bile acid biosynthesis [1]. This is due to the endogenous fermentation of ingested carbohydrates, particularly sugars, by certain bacteria and yeast within the body. Ethanol, regardless of its source, is absorbed throughout the gastrointestinal tract, where two important enzymes are involved in ethanol metabolism. Alcohol dehydrogenase (ADH) oxidizes ethanol into acetaldehyde, and then aldehyde dehydrogenase (ALDH) further catalyzes the conversion of the toxic acetaldehyde to acetate [2]. Therefore, ethanol is mostly broken down before intoxication in healthy individuals. However, there is evidence demonstrating that some people might have abnormal blood ethanol levels even if they did not consume alcoholic beverages. There is a condition called “auto-brewery syndrome”, where significant fermentation of ingested food causes a noticeable level of ethanol to be produced without alcohol consumption [3]. Although this syndrome is extremely rare and was found in patients with diabetes and hepatic diseases, other conditions like prolonged antibiotic use, a carbohydrate-rich diet and/or improper liver enzyme activity, particularly ALDH, might also cause this condition. Apart from endogenous mechanisms that might increase circulating ethanol levels in the body, difficulties in eliminating consumed alcoholic beverages could also cause trouble for people. For example, some people in Eastern Asia suffer from a specific condition called “Asian flush syndrome” due to aldehyde dehydrogenase 2 deficiency in their bodies. Studies showed that approximately 35–50% of East Asians showed this characteristic physiological response after drinking alcohol [4]. AFS could have serious health effects such as nausea, headache, dizziness and increased heart rate [5,6]. Intolerance to ethanol might also cause hangovers, and chronic drinking increases the risks of alcoholic liver disease, inflammation, cognitive impairment and even cancer [7,8,9]. Therefore, even if we drink reasonably, other factors like the disturbance of the microbiome and genetic deficiency could cause undesired sequelae after consuming alcohol. While there is no cure for these conditions yet, it is possible that we can make use of effective supplementation to prevent the influence of alcohol consumption to minimize the incidence of hangover-like symptoms. Emerging research into plant-based molecules shows promise for improving hangover symptoms [10]. Products like flush prevention pills can aid detoxification for more comfortable social drinking. Alcohol degradation protein (ADP) is an animal-based product consisting of ADH and ALDH extracted from *Bos taurus*. It is a patented product aimed to promote the breakdown of both alcohol and acetaldehydes, which could cause adverse feelings after drinking. In our study, we tested the toxicity and effectiveness of ADP in counteracting the acute toxic effect of ethanol. The efficacy of ethanol elimination was determined. The underlying mechanism was also analyzed to better understand the benefits of using ADP in social settings when drinking is unavoidable.

## 2. Materials and Methods

### 2.1. Chemicals and Reagents

Alcohol degradation protein (ADP) is a mixture of ADH and ALDH extracted from *Bos taurus*. It was kindly provided by Alcolear Limited. Absolute ethanol (>99.8%) was purchased from Anaqua Chemicals Supply, USA. King drink (Neptunus, Shenzhen, China) was purchased from a local drugstore. As described in the product manual, it is an extraction of oyster, and the main ingredients include oyster essence, vitamin C, L-cysteine, taurine, calcium pantothenate, starch and sucrose. The active ingredient of king drink is taurine (approximately 8.465 mg/g). HO-1 (#82551), Nrf2 (#12721) and β-actin (#12262) primary antibodies were purchased from Cell Signalling Technology, Danvers, MA, USA. Dulbecco’s modified Eagle’s medium (HG DMEM), Fetal bovine serum (FBS) and penicillin streptomycin (PS) were purchased from Life Technologies Corporation, Carlsbad, CA, USA. An Ethanol Colorimetric Assay Kit, Aldehyde Dehydrogenase (ALDH) Activity Assay Kit and Alcohol Dehydrogenase (ADH) Activity Assay Kit were purchased from Elabscience Biotechnology Co. Ltd., Wuhan, China.

### 2.2. Cell Culture

The HepG2 cell line was cultured in high glucose DMEM supplemented with 10% FBS and 1% PS in an incubator with a stabilized 5% carbon dioxide in the air at 37 °C.

HepG2 cells were seeded in 96-well plates at 1 × 10^5^ cells/mL. The cells were treated with different concentrations of ADP extract (0.125–2 mg/mL) with 5% ethanol for 24 h. Cell viability was determined using an MTT (3-(4,5-dimethyl-2-thiazolyl)-2,5-diphenyl-2-H-tetrazolium bromide) assay after 24 h incubation of 5% ethanol and ADP extract [11,12]. In brief, 0.5 mg/mL MTT solution was incubated with the cells for 4 h. After incubation, the MTT solution was discarded, and DMSO was added to dissolve the formazan crystals. The absorbance of dissolved formazan was detected using a microplate reader at 450 nm (CLARIOstar Plus, BMG LABTECH, Ortenberg, Germany).

### 2.3. Zebrafish Animal Model

Wild-type zebrafish AB strain was purchased from the Zebrafish International Resource Centre (ZIRC; University of Oregon, Eugene, OR, USA) and reared in a circulating aquatic tank system. Zebrafish maintenance and embryo collection were conducted according to established protocols [13]. Briefly, the aquarium temperature was maintained at 26 ± 1 °C, the pH ranged from 6.5 to 8.5 and the light cycle was 14:10 h light/dark. Fish were fed with dried flake food twice and brine shrimp once per day. Zebrafish embryos were collected through natural spawning by employing ‘egg traps’ placed at the bottom of the spawning or maintenance tanks as previously described [14]. The experimental procedures of this study were ethically approved by the Department of Health of the Government of the Hong Kong SAR, China [Ref:(23–86) in DH/HT&A/8/2/8 Pt.5].

#### Zebrafish Anti-Hangover Test

The 5 days post fertilization (dpf) zebrafish larvae received 2% EtOH exposure for 2 h, followed by a 1 h ADP treatment without the presence of EtOH. After 1 h, the zebrafish larvae were exposed to 3 alternating light/dark cycles. Each cycle consisted of 5 min of the dark condition followed by 5 min of the light condition. The dark/light test lasted for a total of 30 min. The locomotor responses of zebrafish were monitored and recorded using Noldus EthoVision XT^®^ 17 tracking software (Noldus, Wageningen, The Netherlands). Locomotor activity analysis was performed as described in a previous study [15]. The average distance traveled (mm), average swimming velocity and average cumulative movement duration during the three dark phases were analyzed.

### 2.4. Acute Ethanol Metabolism Assay

This study was approved and carried out in accordance with the recommendations of the Code of Ethics for Teaching or Research Involving Animal Subjects, Hong Kong Polytechnic University (ASEARS No. 23-24/714-FSN-R-STUDENT). Sprague Dawley rats (male, 6 weeks old and ~200–220 g body weight) were used in this study. Animals were housed under 12 h light/dark cycles and at 22 ± 2 °C and were acclimatized for 7 days. They were fed with a standard diet and water ad libitum. Prior to the experiment, the SD rats were fasted with unlimited access to water for 18 h to avoid any interference with the absorption of ethanol due to feed intake. The volume of the experimental substances to be orally administered to the animal was calculated based on the body weight measured prior to the start of the test. The experimental substances were administered orally 30 min before ethanol administration. Distilled water was administered to the normal control group and the ethanol-alone group instead of the experimental substance. An amount of 3 g/kg (30%) of ethanol was orally administered to all groups except the normal control group.

### 2.5. Biochemical Analysis

To analyze the biochemical changes in the blood caused by the administration of ethanol and experimental substances, blood sampling was conducted at 0, 0.25, 0.5, 1, 2, 4 and 7 h after ethanol administration. The collected blood was centrifuged at 3000 rpm for 10 min. The plasma, liver and cecum content were collected and stored at −80 °C until analysis. The levels of ethanol, alcohol dehydrogenase (ADH) and aldehyde dehydrogenase (ALDH) were analyzed by using commercial kits (Elabscience, Wuhan, China) according to the manufacturer’s protocol using a CLARIOstar Plus microplate reader at 450 nm (BMG LABTECH, Ortenberg, Germany).

### 2.6. Western Blot

SD rats were euthanized after drawing blood, and their livers were extracted. The liver was weighed and stored at −80 °C until analysis. Liver samples were lysed according to standard Western blot protocol. Equal amounts of proteins were separated by SDS–polyacrylamide gel electrophoresis, transferred onto a polyvinylidene fluoride membrane and blocked with a 5% BSA blocking buffer. The membrane was incubated with primary antibodies diluted 1:1000 in a blocking buffer. Bands were detected by enhanced chemiluminescence staining (Life Technologies, Carlsbad, CA, USA) and visualized using a BIO- RAD ChemiDocTM XRS + System (Bio-Rad, Hercules, CA, USA).

### 2.7. Histological Analysis

Small sections of fresh liver tissues were fixed in 10% formalin, followed by dehydration, and embedded in paraffin wax. Tissue sections (4 μm) were stained with hematoxylin and eosin (H&E, Elabscience Biotechnology Co., Ltd., Wuhan, China) for examination of liver damage under optical microscopy (Olympus, Pennsylvania, PA, USA). The infiltration of cytokines was observed to estimate the level of inflammation in the liver.

### 2.8. Statistical Analysis

Values are presented as means ± S.D. All data were analyzed using GraphPad Prism (Version 9.0, GraphPad Software Inc., San Diego, CA, USA). One-way ANOVA was used to compare multiple treatment groups, followed by Dunnett’s test for pairwise comparisons. The numbers of rats used are described in the corresponding figure legends. All experiments were repeated three or more times. Two-sided *p* < 0.05 was considered statistically significant.

## 3. Results

### 3.1. ADP Was Non-Toxic and Protected HepG2 Cells from Ethanol Challenges

**Figure 1:**
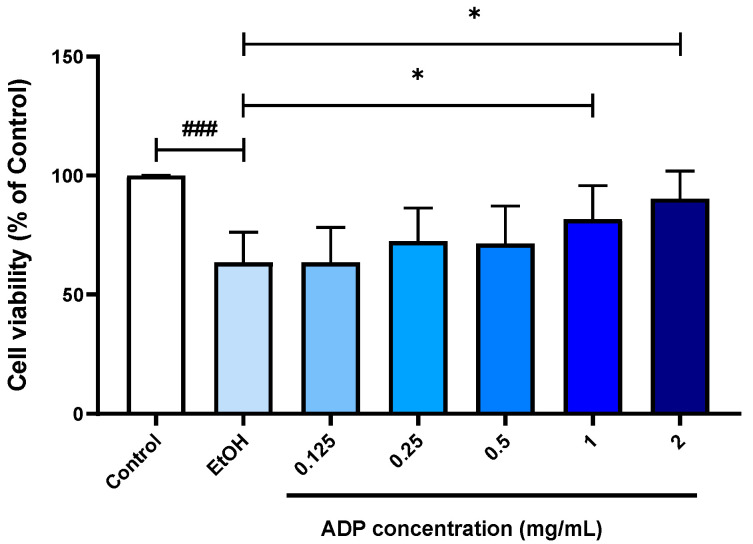
Protective effect of ADP on HepG2 cells challenged with EtOH.

We first evaluated the safety and hepatoprotective effect of ADP using an in vitro model. After treatment with 5% ethanol, the relative cell viability was significantly reduced to 63.57%. However, most of the cells co-treated with ADP had higher cell viability when compared to the ethanol-challenged cell. The protective effect of ADP was in a dose-dependent manner, with significant differences in the cells treated with 1 mg/mL (81.77%) and 2 mg/mL of ADP (90.23%) (Figure 1). Therefore, we believe that ADP was non-toxic in HepG2 cells and could protect against ethanol-induced cytotoxicity.

HepG2 cells were challenged with 5% EtOH in the presence of different concentrations of ADP. Cell viability was measured 24h after ethanol was challenged. Data are presented as mean ± SD (n = 8). The percentage of viable cells was calculated with untreated control cells. The significance of the difference between EtOH and ADP treatment was compared vs. untreated cells at ### *p* < 0.001 and vs. EtOH at * *p* < 0.05.

### 3.2. ADP Prevented Hangover-like Symptoms in Zebrafish Model

**Figure 2:**
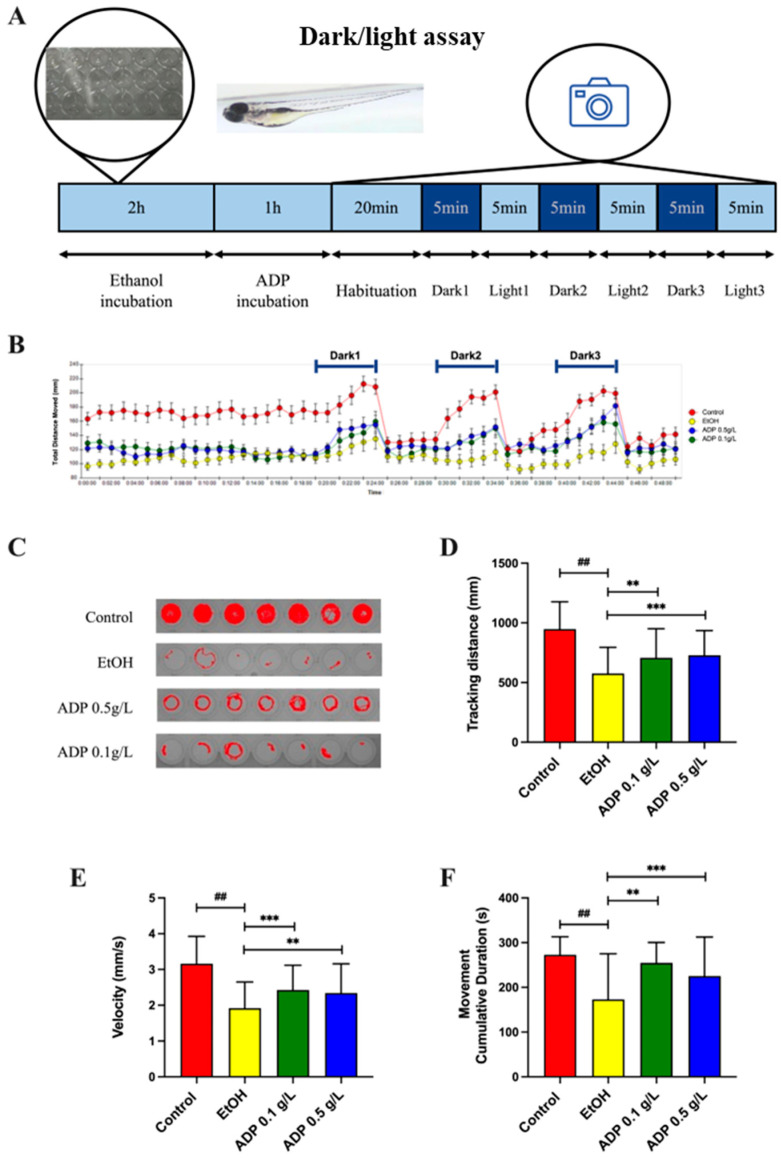
ADP showed an anti-hangover effect in zebrafish larvae. (**A**) Schematic illustration of the design of the zebrafish dark/light assay. The zebrafish larvae received a 2h treatment of 2% EtOH. Then, the zebrafish larvae were exposed to ADP without 2% EtOH for 1 h. After 1 h, the zebrafish larvae were exposed to 3 alternating dark/light cycles (5 min dark–5 min light). Analysis was performed on the average parameters of the three dark phases. (**B**) The total moving distance of zebrafish larvae during the analysis period. (**C**) Representative swimming tracks of zebrafish during one of the dark phases. (**D**–**F**) Quantitative analysis of average tracking distance, swimming velocity and cumulative moving duration in the three dark phases. All data are presented as mean ± SD (n = 24). The significance of the difference was compared vs. control at ## *p* < 0.01 and vs. EtOH at ** *p* < 0.01 and *** *p* < 0.001 in (**D**–**F**).

The behavior of zebrafish after ethanol treatment could simulate the physiological responses after ethanol consumption [15]. Similar to previous studies, our results showed that 2% ethanol reduced the average swimming distance (from 947.94 mm in the control to 576.24 mm in the EtOH group), average swimming velocity (3.16 mm/s vs. 1.92 mm/s) and average cumulative activity time (272.78 s vs. 173.14 s) during the three dark phases. The ethanol-induced decrease in zebrafish activities was significantly alleviated by ADP treatment in both dosages (Figure 2D–F, *p* < 0.01 and *p* < 0.001, respectively). This recovery of locomotor activity after ADP treatment demonstrated that ADP could prevent hangover-like symptoms and low activity in zebrafish.

### 3.3. ADP Promoted Ethanol Metabolism without Altering Liver Functions in SD Rat Model

**Figure 3:**
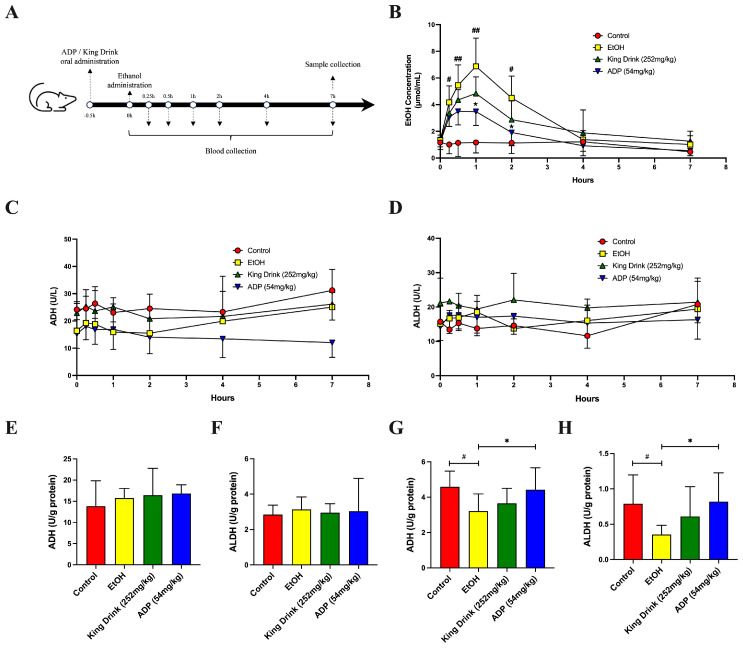
ADP accelerated ethanol elimination after acute ethanol administration. (**A**) Schematic illustration of the design of ethanol metabolism assay. SD rats received an acute administration of 30% EtOH. ADP and King Drink were pre-administered to respective treatment groups 30 min before EtOH administration. Blood was collected through the tail vein at different time points. (**B**) Blood ethanol concentration curve following acute intake of EtOH. (**C**,**D**) Blood ADH and ALDH activity over the 7 h time course. (**E**,**F**) Liver ADH and ALDH activity at 7 h after ethanol administration. (**G**,**H**) ADH and ALDH activity in cecum content of animals 7 h after ethanol administration. All data are presented as mean ± SD (n > 8). The significance of the difference in treatment groups was compared vs. control at ^#^ *p* < 0.05; ^##^ *p* < 0.01; and vs. EtOH at * *p* < 0.05 in (**B**,**G**,**H**).

After testing the effect of ADP in the cell and non-mammal model, we also tested the efficacy of ADP in the SD rat model. Thirty-minute pre-administration of ADP could significantly decrease blood ethanol concentration at 1 h after ethanol administration. The level of blood ethanol in the ADP treatment group was nearly half of the level in the EtOH group (Figure 3B, *p* < 0.05). Although ADP has a similar effect when compared to the positive control King Drink group, it seems ADP was more efficient in ethanol clearance from 1 h onwards when compared with the King Drink treatment (*p* < 0.05 in ADP vs. no significance in the King Drink group). Since ADP is an enzymatic product extracted from the liver, we tested whether ADP administration changed its activity in blood. Interestingly, blood ADH and ALDH activity were not affected either by ethanol or ADP administration. Subsequently, we determined the activity of these two enzymes in the liver and cecum content. Results showed that the activity of ADH and ALDH in the liver was also not affected by acute ethanol administration. However, ADH and ALDH activity were significantly increased in cecum content in rats at 7.5 h after ADP intake (Figure 3G,H, *p* < 0.05). This demonstrates that ADP might accelerate ethanol degradation inside the GI tract prior to being absorbed into the blood and liver.

### 3.4. ADP Exerted Potential Antioxidant Properties by Inhibiting Acute Stimulation of HO-1 Expression in SD Rat Model

**Figure 4:**
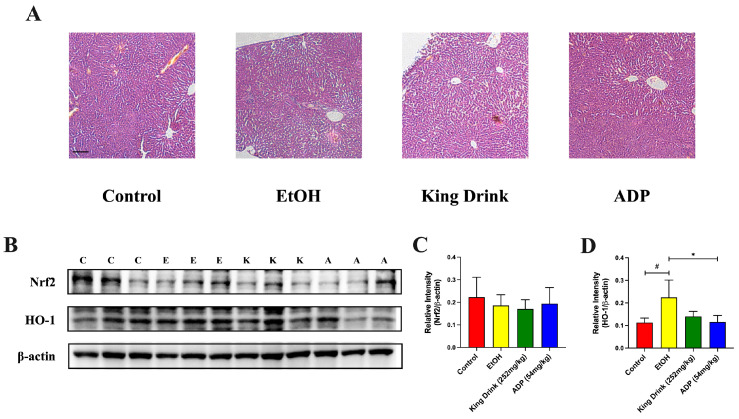
ADP prevented over-activation of HO-1 after acute ethanol administration. (**A**) Effects of ADP on histopathological changes in liver tissues after acute ethanol administration. Bar = 50 μm. (**B**) Representative Western blot bands for protein expressions in liver of animals pretreated with ADP and acute ethanol ingestion. C: control; E: EtOH; K: King Drink; and A: ADP group. (**C**,**D**) Statistical analysis of Nrf2 and HO-1 protein expression in liver. All data are presented as mean ± SD (n = 3). The significance of difference in treatment group was compared vs. control at # *p* < 0.05 and vs. EtOH at * *p* < 0.05 in (**D**).

In addition, we determined whether ADP would have toxicity or provide protection in an animal model. Histological slice demonstrated that no abnormal morphologies were observed in all treatment groups. Western blot showed that acute administration of ethanol significantly lowered HO-1 expression in the liver (Figure 4D, *p* < 0.05). However, neither ADP nor King Drink could restore Nrf2 expression. Consolidating the above results, we believe that ADP has two possible anti-alcoholic mechanisms. The first one should be a direct oxidation of ethanol inside the GI tract, and the second one is to inhibit the over-activation of the HO-1 enzyme to prevent excessive consumption of HO-1 in the sub-acute stage after ethanol consumption.

## 4. Discussion

Alcohol is a famous beverage both for leisure and social functions. According to statistics, the sales of alcoholic beverages has increased by 54% in 2020 worldwide [16]. After oral consumption of alcohol, it is absorbed by the mucosa of the gastrointestinal tract and subsequently through the portal vein to the liver [17]. More than 80% of the ethanol will undergo oxidation by the enzyme ADH, forming a toxic acetaldehyde and nicotinamide adenine dinucleotide (NADH). The toxic acetaldehyde is further oxidized by ALDH to produce acetate, which enters the Krebs cycle to form carbon dioxide and water, and is eliminated from the body [18]. Both enzymes are mainly found in the liver, although they can also be detected in the gastric mucosa and small intestine. Therefore, it is possible that increasing the activities of these enzymes could help accelerate the metabolism and elimination of ethanol from the body. In the current study, the patented alcohol degradation protein (ADP) extracted from *Bos taurus* was used to determine if it will provide beneficial physiological effects towards acute ethanol challenge in various models. Apart from the in vitro HepG2 cellular model, we used two different animal models, including zebrafish and an SD rat model, to evaluate the effect of ADP under ethanol challenge. The utilization of zebrafish in exploring the anti-hangover effects of ADP following exposure to ethanol is based on the genetic similarities between zebrafish and humans, as well as their transparency and rapid development [19]. Behavioral tests have proven invaluable in understanding how zebrafish respond to alcohol [20]. In our study, ADP improved locomotor activities after ethanol treatment. The increased moving duration and swimming velocity strongly suggested that ADP could prevent hangover-like symptoms such as fatigue, drowsiness, sleepiness and weakness [21]. After ADP treatment for around 1–2 h, where ethanol commonly peaked in the body after consumption [22], the movement of zebrafish was significantly higher. These data coincide with our SD rat model. Pre-administration of ADP significantly lowered blood ethanol concentration at 1 h and 2 h time points. Interestingly, our initial hypothesis of ADP increasing ADH and ALDH activities in blood and liver was not detected in the SD rat model. However, ADH and ALDH activities were significantly increased in cecum content, suggesting that ADP might spontaneously react with the ethanol ingested inside the GI tract and ultimately achieve fast elimination.

Regarding chronic liver diseases around the world, ethanol could induce chronic inflammation and oxidative stress in the liver due to the metabolism of ethanol by the microsomal-oxidizing system. In the process, a large amount of reactive oxygen species (ROS) is produced by the cytochrome P450 enzyme CYP2E1. These ROS could possibly cause lipid peroxidation, leading to hepatic inflammation and even steatosis [23]. In our study, ADP also decreased the stimulated HO-1 protein expression caused by ethanol administration. HO-1 is an inducible enzyme that is readily activated by a variety of stimuli, such as oxidative stress. Acute ethanol stimulation demonstrated an increased reactivity in the early stages. However, excessive consumption of HO-1 might occur during chronic stress, leading to a decrease in its levels [24]. ADP treatment lowered the expression of HO-1 after acute ethanol challenges, suggesting that the ADP also processes certain antioxidation effects, which could be beneficial to use before ethanol consumption to prevent unpleasant feelings. However, the current study had limitations because we only tested one single dose of ethanol (approximately equivalent to consuming 30 g of 30% ethanol in humans). It will provide more scientific evidence for the effect of ADP on anti-alcoholic symptoms if we could have data on heavy drinking or chronic drinking. We also believe that there could be species differences between animals and humans, which leads us to our future planning on designing clinical trials.

## 5. Conclusions

ADP is an enzymatic product consisting of ADH and ALDH extracted from *Bos taurus*. It was demonstrated that ADP could effectively prevent hangover-like symptoms and lower blood ethanol concentration in various animal models. The mechanism of ADP was possibly related to its direct interaction with ingested ethanol inside the GI tract, which accelerated the degradation of ethanol and prevented it from entering the hepatic system.

## Data Availability

The original contributions presented in the study are included in the article material, further inquiries can be directed to the corresponding author.
